# Isotypes of Epstein-Barr Virus Antibodies in Rheumatoid Arthritis: Association with Rheumatoid Factors and Citrulline-Dependent Antibodies

**DOI:** 10.1155/2015/472174

**Published:** 2015-04-27

**Authors:** Marie Wulff Westergaard, Anette Holck Draborg, Lone Troelsen, Søren Jacobsen, Gunnar Houen

**Affiliations:** ^1^Department of Autoimmunology and Biomarkers, Statens Serum Institut, Artillerivej 5, 2300 Copenhagen, Denmark; ^2^Department of Clinical Immunology, Rigshospitalet, Copenhagen University Hospital, Blegdamsvej 9, 2100 Copenhagen, Denmark; ^3^Department of Rheumatology, Rigshospitalet, Copenhagen University Hospital, Blegdamsvej 9, 2100 Copenhagen, Denmark

## Abstract

In order to study the humoral immune response against Epstein-Barr virus (EBV) in patients with rheumatoid arthritis (RA) and to compare it with the two major autoantibody types in RA, plasma samples from 77 RA patients, 28 patients with systemic lupus erythematosus (SLE), and 28 healthy controls (HCs) were investigated by enzyme-linked immunosorbent assays (ELISA). Increased percentages of positives and concentrations of IgG/IgA/IgM antibodies against the latent EBV nuclear antigen-1 (EBNA-1) were observed in RA patients compared to SLE patients and HCs. Increased concentrations and percentages of positives of IgG/IgA/IgM against the early lytic EBV antigen diffuse (EAD) were also found in RA patients compared to HCs but were highest in SLE patients. Furthermore, associations between the elevated EBNA-1 IgA and EBNA-1 IgM levels and the presence of IgM and IgA rheumatoid factors (RFs) and anti-citrullinated protein antibodies (ACPAs, IgG) and between elevated IgA concentrations against EAD and the presence of RFs and ACPAs in RA patients were found. Thus, RA patients had elevated antibodies of all isotypes characteristic of latent EBV infection (whereas SLE patients had elevated antibodies characteristic of lytic EBV infection). Notably, for IgM and IgA (but not IgG), these were associated with the presence of characteristic RA autoantibodies.

## 1. Introduction

Rheumatoid arthritis (RA) is a chronic inflammatory systemic autoimmune disease. Worldwide, the prevalence is estimated to be about 0.5%–1%, but the incidence and prevalence vary geographically and are 2-3-fold higher in women than in men. The disease is characterised by inflamed joints and the production of autoantibodies, for example, rheumatoid factors (RFs) and anti-citrullinated protein antibodies (ACPAs). The etiology of the disease is suggested to be a combination of environmental exposures and gene-environment interactions, but the exact cause is still unknown [1, 2]. One environmental factor may be the human herpesvirus, Epstein-Barr virus (EBV).

EBV is one of the most common viruses found in humans and is believed to infect approximately 95% of the worldwide population before an age of 40 years [3]. EBV is transmitted through saliva and infects and replicates in epithelial cells and B cells. The primary infection with EBV is mostly asymptomatic during childhood, but during adolescence it can cause infectious mononucleosis [4].

After primary infection EBV persists latently in memory B cells, where the only protein expressed is the Epstein-Barr virus nuclear antigen 1 (EBNA-1), which is responsible for maintaining viral DNA during the cell cycle and has a characteristic Gly-Ala repeat region with a presumed role in immune evasion by EBV. Occasionally, the virus reactivates and enters the lytic stage expressing genes promoting viral replication and release of virions [4, 5].

The EBV protein, early antigen diffuse (EAD) is expressed during the early lytic stage of EBV's lifecycle. It is a DNA polymerase accessory protein and is required for initiating lytic viral replication. The presence of EAD antibodies indicates initiation of viral replication [6, 7].

Cellular immunity is essential for controlling EBV infection, but the humoral immune response is also activated during EBV infection and different serological profiles can reflect the infection status/history. Viral-capsid antigen (VCA) and EAD IgM and IgG antibodies are produced during primary infection and EBNA-1 IgG antibodies are produced later in the infection. VCA IgM antibodies disappear after convalescence while VCA IgG antibodies and EBNA-1 IgG have lifelong persistence [8, 9]. IgA against EBV antigens have to our knowledge not been investigated before in RA patients.

Several studies have shown an elevated humoral and cellular anti-EBV immune response in RA patients, indicating that the virus may be associated with the autoimmune dysfunction in patients with RA [10–14]. Elevated antibody levels have been found against EBV proteins, such as VCA, EAD, early antigen restricted (EAR), and EBNA-1, in RA patients compared to healthy controls and disease controls [10–13]. In addition, RF positive RA patients have elevated EBNA-1 antibody concentrations compared to RF negative RA patients [10]. These studies have mainly focused on EBV IgG antibodies.

To obtain a detailed picture of the immune response to antigens representing the latent and lytic phases of the EBV life cycle and in order to investigate possible epithelial involvement we studied the occurrence of EBNA-1 and EAD antibodies (IgM, IgG, and IgA) in RA patients and control groups. Moreover, we looked for a possible association between EBV antibodies and the RA-characteristic autoantibodies RFs and ACPAs. Such an association would strengthen a theory of EBV as a major etiological agent in RA.

## 2. Patients and Methods 

### 2.1. Patients and Controls

All patients fulfilled internationally accepted classification criteria for the autoimmune diseases investigated [15, 16]. Consents for the studies were obtained from all patients in accordance with the protocol approved by the Scientific-Ethical Committee of the Capital Region of Denmark (number HA-2007-0114).

Plasma samples were obtained from 77 RA patients and 28 SLE patients attending the Department of Rheumatology at Copenhagen University Hospital in Denmark.

Plasma samples from 28 healthy controls (HCs) were obtained with consent from volunteers at SSI, Copenhagen, Denmark.

Clinical characteristics of all patients and controls are outlined in Table 1. SLE patients and HCs have previously been reported on in the study by Draborg et al. [17] as well as RA patients in the study by Troelsen et al. [18].

### 2.2. ELISA

#### 2.2.1. EBV Antibodies

ELISA was performed as previously described by Draborg et al. [6]. Briefly, dilutions and washes were performed in TTN (0.025 M Tris, 0.5% Tween 20, 0.15 M NaCl, pH 7.5). EBV EAD (Prospec protein specialist, Ness-Ziona, Israel) and EBV EBNA-1 ((whole protein) MyBioSource, San Diego, California, USA) diluted in carbonate buffer to a concentration at 1 *μ*g/mL and 0.5 ug/mL, respectively, were applied to a 96-well microtiter polysorp plate (Nunc, Fisher Scientific Biotech Line A/S). The antigen was incubated overnight (O/N) at 4°C followed by 30 min blocking of unoccupied sites with TTN. Plasma (1 : 100 or 1 : 20 for detecting IgG antibodies or IgA/IgM antibodies, resp.) was incubated for one hour. The wells were washed before incubation with AP-conjugated goat anti-human IgG/IgA/IgM secondary antibodies (Sigma) (1 : 2000). The wells were washed again before adding* p*-nitrophenylphosphate (*p*-NPP) (1 mg/mL) diluted in AP substrate buffer. The absorbance was measured at wavelength 405–650 nm (Thermo-Max Microplate Reader, Molecular Devices).

Each sample was tested in duplicates in both coated wells and noncoated wells. For each plate, a standard curve was included using a high titre sample for normalisation of the absorbance. A sample was considered positive for EAD or EBNA-1 antibodies if the antibody binding was higher than the cut-off value (14.53, 2.98 and 22.78 U/mL, regarding EAD IgG, EAD IgA, and EAD IgM antibodies, resp., and 1.50, 7.70, and 8.27 U/mL, regarding EBNA-1 IgG, EBNA-1 IgA, and EBNA-1 IgM antibodies, resp.). The cut-off values were determined from the HCs (without outliers (determined with Grubb's test)) using the mean + 2 × SD.

For some experiments, a mosaic EBNA-1 protein without the Gly-Ala repeat region was used (results not shown).

#### 2.2.2. Autoantibodies

ACPAs were determined by a commercial ELISA kit (Euro-Diagnostica, Malmö, Sweden), measuring IgG antibodies against cyclic citrullinated peptides (CCPs). Instructions by the manufacturer were followed.

IgM and IgA RFs were determined by ELISA using polystyrene plates (Maxisorp) coated with purified IgG. Peroxidase-conjugated F(ab)2 rabbit immunoglobulin against human IgM and IgA (DAKO, Copenhagen, Denmark), respectively, was used as detecting antibodies [19].

### 2.3. Statistics

Statistical analyses were carried out using GraphPad Prism software 5 (GraphPad Prism software, Inc., La Jolla, CA, USA). Comparison of antibody concentrations between patients and controls was performed using a Mann-Whitney test. Comparison of paired samples was performed using a Wilcoxon test. Statistical significant differences are indicated with ∗, ∗∗, ∗∗∗, or ∗∗∗∗ for *P* values less than 0.05, 0.01, 0.001, or 0.0001, respectively. Data are presented with the mean ± SEM.

## 3. Results 

### 3.1. Early Antigen Diffuse (EAD)

**Figure 1:**
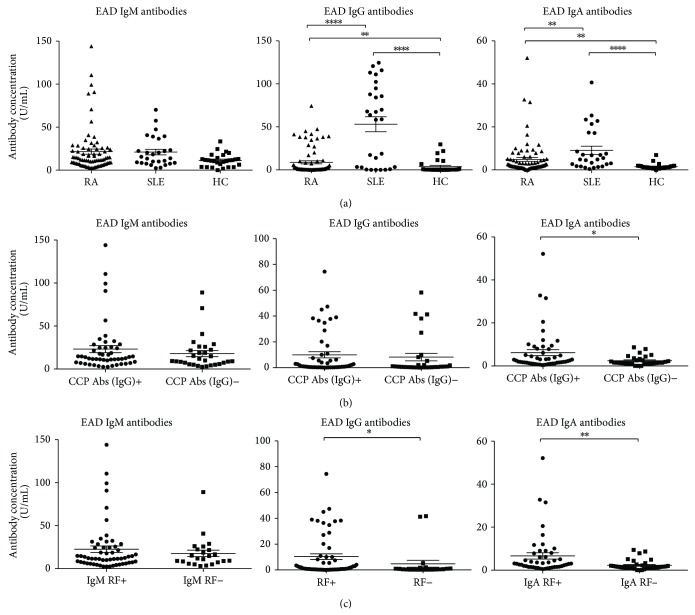
Scattergrams showing individual levels of EAD IgG/IgM/IgA in plasma from RA patients, SLE patients, and HCs and individual levels of EAD IgG/IgM/IgA from RA patients divided into CCP IgG positives and negatives and divided into IgA RF and IgM RF positives and negatives. (a) EAD IgM/IgG/IgA levels. RA patients (*n* = 77), SLE patients (*n* = 28), and HCs (*n* = 28). (b) EAD IgM/IgG/IgA levels sorted into CCP IgG positives (*n* = 49) and CCP IgG negatives (*n* = 28). RA patients total (*n* = 77). (c) EAD IgA levels sorted in IgA RF states, IgA RF positives (*n* = 44) and IgA RF negatives (*n* = 33). EAD IgG levels sorted in RF states, RF positives (*n* = 56) and RF negatives (*n* = 21). EAD IgM levels sorted in IgM RF states, IgM RF positives (*n* = 54) and IgM RF negatives (*n* = 23). RA patients total (*n* = 77). Groups were compared using the Mann-Whitney test. Data are presented as mean ± SEM. Concentrations of antibodies are presented in arbitrary units. Statistical significant differences are indicated with ∗, ∗∗, ∗∗∗, or ∗∗∗∗ for *P* values less than 0.05, 0.01, 0.001, or 0.0001, respectively.

An indirect ELISA was used to measure the amount of EAD IgM/IgG/IgA in plasma samples from 77 RA patients, 28 SLE patients (disease controls), and 28 HCs (Figure 1(a)). No significant differences were observed between the groups regarding EAD IgM levels, although RA and SLE patients appeared to have slightly higher levels. However, significant differences in EAD IgG and EAD IgA levels were seen between RA patients and HCs, between SLE patients and HCs, and between RA patients and SLE patients (*P* values of 0.0011, <0.0001, and <0.0001, resp., for EAD IgG and *P* values of 0.0016, <0.0001, and 0.0056, resp., for EAD IgA) (Figure 1(a)). The levels of EAD IgG and IgA were highest in SLE patients and lowest in HCs.

By use of cut-off values determined from HCs, the percentages of EAD positives were calculated. RA patients had a higher percentage of antibody positive of EAD IgM/IgG/IgA than HCs (Table 2). SLE patients had a higher percentage of positives of all 3 EAD antibody isotypes than both RA patients and HCs.

To examine a possible correlation between the two types of RA-characteristic autoantibodies and the lytic EBV protein EAD antibodies, the results for RA patients were sorted in CCP antibody positives and negatives (Figure 1(b)) and RF IgM/IgA positives and negatives (Figure 1(c)). When sorted by CCP antibody status, a significant difference was seen for EAD IgA but not for IgM or IgG (Figure 1(b)). EAD IgA levels were higher in the group with CCP IgG (*P* = 0.048). When sorted by RF status, significant differences were seen for EAD IgA and IgG (Figure 1(c)). EAD IgA levels were highest in the group with RF IgA (*P* = 0.001) and EAD IgG levels were highest in the RF positive group (RF IgM and/or IgA) (*P* = 0.032). No other significant differences were seen, when these results were sorted by RF status (IgM and/or IgA).

### 3.2. Epstein-Barr Virus Nuclear Antigen-1 (EBNA-1)

**Figure 2:**
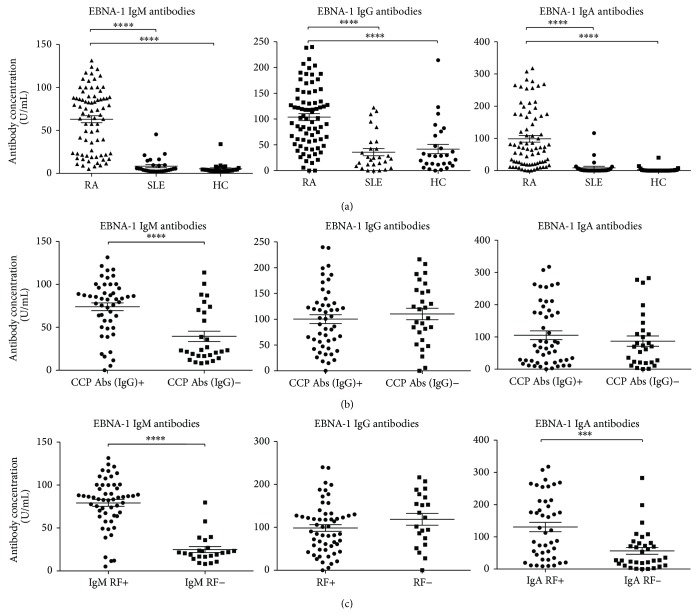
Scattergrams showing individual levels of EBNA-1 IgG/IgM/IgA in plasma from RA patients, SLE patients, and HCs and individual levels of EBNA-1 IgG/IgM/IgA from RA patients divided into CCP IgG positives and negatives and divided into IgA RF and IgM RF positives and negatives. (a) EBNA-1 IgM/IgG/IgA levels. RA patients (*n* = 77), SLE patients (*n* = 28), and HCs (*n* = 28). (b) EBNA-1 IgM/IgG/IgA levels sorted into CCP IgG positives (*n* = 49) and CCP IgG negatives (*n* = 28). RA patients total (*n* = 77). (c) EBNA-1 IgA levels sorted in IgA RF states, IgA RF positives (*n* = 44) and IgA RF negatives (*n* = 33). EBNA-1 IgG levels sorted in RF states, IgM or IgA RF positives (*n* = 56) and RF negatives (*n* = 21). EBNA-1 IgM levels sorted in IgM RF states, IgM RF positives (*n* = 54) and IgM RF negatives (*n* = 23). RA patients total (*n* = 77). Groups were compared using the Mann-Whitney test. Data are presented as mean ± SEM. Concentrations of antibodies are presented in arbitrary units. Statistical significant differences are indicated with ∗, ∗∗, ∗∗∗, or ∗∗∗∗ for *P* values less than 0.05, 0.01, 0.001, or 0.0001, respectively.

Since EBNA-1 is the only protein expressed by EBV in the latency I stage of its life cycle [4, 20], it was decided to investigate EBNA-1 antibodies in plasma from RA patients. Plasma from 77 RA patients, 28 SLE patients, and 28 HCs was tested for EBNA-1 IgM/IgG/IgA by ELISA (Figure 2(a)). A large and highly significant difference in EBNA-1 IgM/IgG/IgA levels between RA patients and HCs and between RA patients and SLE patients (*P* < 0.0001 for all 3 antibody isotypes) was observed. In contrast, no significant difference was observed between SLE patients and HCs for EBNA-1 IgM/IgG/IgA levels (Figure 2(a)).

Cut-off values, determined from HCs, were used to calculate the percentages of positives for EBNA-1 antibodies. RA patients had noticeably higher percentages of positives for EBNA-1 IgA and EBNA-1 IgM levels than both disease controls and HCs but not for EBNA-1 IgG levels (Table 2).

To examine a possible relation between RA-characteristic autoantibodies and the latent EBV-protein EBNA-1 antibodies, the 77 RA patients were sorted into CCP antibody positives and negatives (Figure 2(b)) and IgA/IgM RF positives and negatives (Figure 2(c)).

A significant coherence between the EBNA-1 IgM levels and the CCP antibody state was observed (*P* < 0.0001) (Figure 2(b)). However, there was no difference for EBNA-1 IgG and EBNA-1 IgA levels with regard to CCP antibodies (Figure 2(b)).

Comparison of the EBNA-1 IgA and EBNA-1 IgM levels in the 77 RA patients sorted into IgM and IgA RF positives and negatives, respectively, revealed a significant difference between the two groups for both EBNA-1 IgM and IgA (*P* < 0.0001 and *P* = 0.0005, resp.) (Figure 2(c)). No correlation was seen when dividing RA patients' EBNA-1 IgG levels by any RF isotype positives and negatives (Figure 2(c)) or between IgM or IgA RF (not shown).

### 3.3. Relationships between RFs and ACPAs

**Figure 3:**
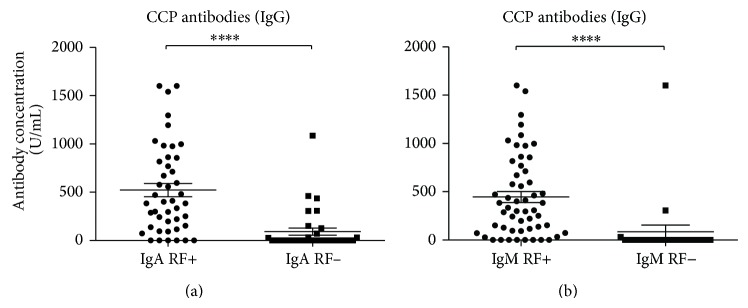
Scattergrams showing CCP antibodies from RA patients divided into IgA or IgM RF positives and negatives. (a) CCP antibodies divided into IgA RF states, IgA RF positives (*n* = 44) and IgA RF negatives (*n* = 33). (b) CCP antibodies divided into IgM RF states, IgM RF positives (*n* = 54) and IgM RF negatives (*n* = 23). RA patients total (*n* = 77). The RF positives and negatives were compared using the Mann-Whitney test. Data are presented as mean ± SEM. Concentrations of antibodies are presented in arbitrary units. Statistical significant differences are indicated with ∗, ∗∗, ∗∗∗, or ∗∗∗∗ for *P* values less than 0.05, 0.01, 0.001, or 0.0001, respectively.

Coherence between RF levels and CCP antibody state was also tested and a highly significant difference was observed in CCP antibody levels when stratified by either IgA or IgM RF state (*P* < 0.0001 for both) (Figures 3(a) and 3(b)).

### 3.4. Relation to Treatments

**Figure 4:**
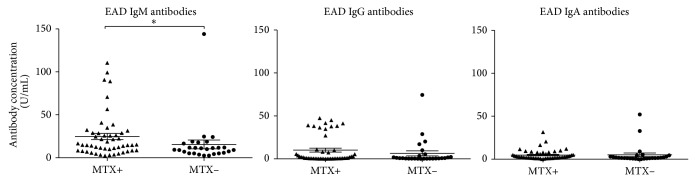
Scattergrams showing individual levels of EAD IgG/IgM/IgA in plasma from RA patients divided into the two groups according to acute Methotrexate (MTX) treatment. EAD IgM/IgG/IgA levels divided into patients on MTX treatment and patients not on MTX treatment. RA patients on MTX (*n* = 50), RA patients not on MTX (*n* = 27), and RA patients total (*n* = 77). The two groups were compared using the Mann-Whitney test. Data are presented as mean ± SEM. Concentrations of antibodies are presented in arbitrary units. Statistical significant differences are indicated with ∗, ∗∗, ∗∗∗, or ∗∗∗∗ for *P* values less than 0.05, 0.01, 0.001, or 0.0001, respectively.

Since immunosuppressive treatments may influence the results of antibody measurements, the influence of immunosuppressive medications such as Methotrexate (MTX), glucocorticoids, NSAIDs, TNF-*α* inhibitors, and DMARDs on both EAD and EBNA-1 antibody levels was investigated. A small but significant (*P* = 0.010) correlation was only observed between elevated EAD IgM levels and the intake of MTX but not for EAD IgG or IgA (Figure 4). No correlations were seen for any drug treatment and EBNA-1 IgM/IgG/IgA (results not shown).

## 4. Discussion

The current study examined the possible relation between EBV and RA. SLE patients were chosen as disease controls, as SLE is a related connective tissue rheumatic disease with distinct clinical and serological characteristics (rather than Sjögren syndrome which has features overlapping with both). The study focused on two antigens characteristic of EBVs different life cycles: the latent EBNA-1 and the early lytic EAD. EBNA-1 is the only essential protein for maintaining the EBV genome during the latency stage of the EBV lifecycle [21] and it is the only protein expressed in the latency stage I.

Elevated antibody concentrations (U/ml) and higher percentages of antibody positives were observed in RA patients, compared to control groups, together with an association between high EBNA-1 antibody concentrations and the presence of the characteristic RA autoantibodies, RFs, and ACPA. More specifically, the percentages of EBNA-1 IgM and IgA were remarkably higher in RA patients compared to SLE patients (disease controls) and HCs and EBNA-1 IgM, IgG, and IgA levels (U/ml) were strongly elevated in RA patients. This indicates an increased humoral response towards EBV in the latency stage in RA patients.

Noticeably, if an individual has been exposed to EBV, EBNA-1 IgG will be measurable throughout the lifetime [22]. Because of the high infection effectiveness of EBV of about 95% of the world population (>40 years old) [3], a similar percentage of EBNA-1 IgG positives were expected in all examined groups consistent with the current observations (Table 2).

In general, IgA and IgM have half-lives in blood of approximately 1 week after production [23, 24]. Therefore, high levels of these isotypes may imply an ongoing humoral immune response [25]. EBNA-1 is expressed at relatively high levels in EBV-infected cells, but the endogenous pathway of presentation of EBNA-1-derived peptides in complex with MHC I on the surface of the cells is restricted due to the Gly-Ala repeat domain in EBNA-1 that prevents degradation in the proteasomes [26]. However, the exogenous pathway presentation of EBNA-1 via MHC II is still effective [27]. This could be a part of the explanation for the present results, indicating a persistent humoral immune response against EBNA-1 released from dying infected cells.

The elevated antibody levels and higher percentages of antibody positives seen in RA patients suggest that the immune surveillance of EBV in RA patients is dysfunctional. This is supported by previous reports that CD8+ T cells specific for lytic EBV proteins are dysfunctional in RA patients [28] and, consequently, an elevated humoral response to EBV has been induced.

The present observations that IgG and IgA levels as well as percentage of antibody positives against the lytic EBV protein EAD are elevated in RA patients compared to HCs also indicate some degree of reactivation of the EBV infection in B cells and epithelial cells.

In agreement with Draborg et al. [6], SLE patients had noticeably higher levels and percentages of antibody positives for both EAD IgG and EAD IgA compared to RA patients, which indicates that reactivation of EBV is more frequent in SLE patients than in RA patients (especially in epithelial cells).

Recent studies have shown that the intake of the immunosuppressive medical Methotrexate (MTX) can lead to increased reactivation of EBV [29]. The elevated levels of EAD IgG and EAD IgA in RA patients did not correlate with patients' intake of MTX in this study. However, a small but significant correlation was observed with elevated EAD IgM levels in patients currently taking MTX at the time where the blood sample was taken, indicating a low degree of EBV reactivation by MTX. Similarly, comparing EAD antibody levels in patients taking other immunosuppressive medications such as glucocorticoids, NSAIDs, TNF-*α* inhibitors, and DMARDs, no correlation was observed (data not shown). Also EBNA-1 antibody levels and the intake of immunosuppressive medications were investigated and again no correlation was observed (data not shown). Altogether, this shows that the present results cannot be explained by drug treatment.

The correlation between RFs and CCP antibodies and the EAD antibody levels was also examined. No correlation between EAD IgM or EAD IgG and the characteristic RA autoantibodies in RA patients was found, while a correlation was observed between elevated EAD IgA levels and IgA RF positivity in RA patients as well as elevated EAD IgA levels in CCP antibody positive RA patients.

No relation between EBNA-1 IgG or IgA levels and CCP antibodies was found. However, an association between the EBNA-1 IgM response and the presence of ACPA was found.

An interesting hypothesis is that EBNA-1 in a citrullinated form could act as a target for ACPAs and that this could be a direct link between EBV infection and the onset or just progression of production of ACPAs. Combined with the observation that citrullinated proteins are found to be abundant in the synovial joints [30, 31], this leads to a theory that ACPA production could play a role in the induction of the inflammation found in the synovial joints of RA patients as suggested by Klareskog et al. [32].

The involvement of exogenous antigens in the production of ACPAs has also been suggested by Pratesi et al. [33]. Observations of the presence of antibodies against citrullinated peptides from EBNA-1 and EBNA-2 in RA patients, while these are almost absent in HCs and disease controls [33, 34], led to speculations of the EBV involvement in the production of ACPA.

This indicates that an antibody response against cells with reactivated EBV infection could have an influence on autoantibody production. In accordance with the observation that several of the ACPAs react with epithelial cells due to their content of a number of the target citrullinated proteins [35], an association could be possible.

No correlation was observed between EBNA-1 IgG levels and the presence of any RF isotype. However, a significant correlation between elevated EBNA-1 IgA levels and the presence of IgA RF as well as association between elevated EBNA-1 IgM levels and the presence of IgM RF in the RA cohort was revealed. The same pattern of coherence between the EBNA-1 antibody levels and the presence of autoantibodies was observed when investigating mosaic EBNA-1 (without the Gly-Ala repeats) antibodies and EBNA-1 antibodies (results not shown). This suggests that the Gly-Ala repeat region does not have a crucial effect on the amount of EBNA-1 antibodies.

The present results are in accordance with a previous study, which observed elevated EBNA antibodies in RF-seropositive RA patients compared to RF-seronegative RA patients [10]. It may be hypothesised from these findings that simultaneous processes are responsible for induction of RFs and EBNA-1 antibodies. These processes could be induced by the recognition and destruction of EBV-infected cells, for example, infected memory B cells, where EBV is in its latency stage I only expressing EBNA-1. Together with the observations that alteration in the glycosylation of IgG from EBV-infected B cells in RA patients can impair the functionality of the IgG [36, 37], a destructed EBV-infected cell releasing EBNA-1 and IgG could be the antigen source responsible for the production of both EBNA-1 antibodies and RFs.

The increased IgM/IgA concentrations against especially the latent EBV protein, EBNA-1, but also IgG/IgA against the lytic EBV protein, EAD, in RA patients compared to HCs are in accordance with previous studies showing elevated levels of IgG against the EBV proteins, EBNA-1, VCA, and EAD in sera from RA patients relative to HCs [10, 11]. Later studies have mainly investigated antibodies against the two lytic EBV proteins VCA and EAD and found them to be elevated in RA patients compared to HCs [12, 13]. Although the present results indicate that the latent EBV infection has a major influence on RA, these previous observations suggested that lytic reactivation of the EBV infection also has an influence on RA.

Finally, the results presented here are in agreement with the polyclonal B cell activation seen in RA (possibly caused by EBV) and the clinical efficacy of B cell-depleting agents in RA [38–41] and are in agreement with previous investigations [42–46]; RFs and ACPAs were found also in this RA cohort to be correlated.

To summarise, we found an association between EBNA-1 antibodies and characteristic autoantibodies in RA patients. As expected, a significant association between anti-CCP antibody levels and presence of IgA RF and IgM RF was observed. Even though the occurrence of antibodies against EBNA-1 in this study was highly associated with occurrence of ACPA and RF antibodies, this study was cross-sectional and further investigations are needed to elucidate the interplay between these factors in the pathogenesis of RA.

## Figures and Tables

**Table 1:** Clinical characteristics of the patients and controls.

	RA	SLE	HC
Number of individuals	77	28	28
Average age (years) (range)	55.5 (27–78)	41.3 (20–81)	36.6 (22–61)
Average disease duration (years) (range)	14.8 (2–41)	—	—
Female (%)	76.6	96.4	96.4
CCP antibody positive (%)	63.6	—	—
RF IgM positive (%)	70.1	7.1	—
RF IgA positive (%)	57.1	25	—
Average hsCRP (mg/L) (range)	11.6 (0.3–69.9)	5.1 (1–22)	—
HAQ score (range)	0.82 (0–2.9)	—	—
TSS (range)	86.1 (0–321)	—	—
Methotrexate (%)	64.9	7.1	—
Glucocorticoids (%)	29.9	60.7	—
NSAID (%)	33.8	14.3	—
Anti-TNF-alfa (%)	36.4	—	—

**(a):** 

	EAD Ab positive (%)		

	IgG	IgA	IgM
RA (*n* = 77)	19.5	37.7	27.3
SLE (*n* = 28)	64.3	64.3	28.6
HC (*n* = 28)	10.7	7.1	7.1

**(b):** 

	EBNA-1 Ab positive (%)		
	IgG	IgA	IgM
RA (*n* = 77)	97.4	93.5	97.4
SLE (*n* = 28)	89.3	17.6	32.1
HC (*n* = 28)	96.4	7.1	7.1
